# Care and support for youth living with HIV/AIDS in secondary schools: perspectives of school stakeholders in western Uganda

**DOI:** 10.1186/s12889-020-10143-3

**Published:** 2021-01-06

**Authors:** Emmanuel Kimera, Sofie Vindevogel, Didier Reynaert, Anne-Mie Engelen, Kintu Mugenyi Justice, John Rubaihayo, Jessica De Maeyer, Johan Bilsen

**Affiliations:** 1 Department of Public Health, School of Health Sciences, Mountain of the Moon University, Fort Portal, Uganda; 2 Department of Social Educational Carework & EQUALITY Research Collective, University of Applied Sciences and Arts Gent, Brussels, Belgium; 3 School of Education, Mountains of the Moon University, Fort Portal, Uganda; 4 Department of Public Health, Mental Health and Wellbeing research group, Vrije Universiteit Brussel, Brussels, Belgium

**Keywords:** HIV/AIDS, Qualitative, Youth, HIV/AIDS-competence, Social support

## Abstract

**Background:**

Although schools have been identified as significant settings in the response to the HIV/AIDS pandemic, limited research is available on how they can accommodate Youth Living with HIV/AIDS (YLWHA), especially in resource limited countries. In this study, we explored strategies by school stakeholders (school staff, parents/caretakers, and students) in western Uganda to care for and support YLWHA in their schools.

**Methods:**

The article utilizes data collected between May and October, 2019 from a qualitative inquiry based on focus group discussions and interviews with 88 school stakeholders purposively selected from 3 secondary schools in western Uganda. Textual data was analyzed thematically involving both inductive and deductive coding.

**Results:**

We identified 7 overarching interrelated themes in which participants reported strategies to care for and support YLWHA: counselling and guidance; social support networks and linkages; knowledge and skills; anti-stigma and anti-discrimination measures; disclosure of HIV status; treatment and management of HIV/AIDS; and affirmative actions for YLWHA. Stakeholders’ strategies often differed regarding what was considered appropriate, the approach and who to take lead in supporting YLWHA.

**Conclusions:**

Despite the limited care and support strategies specific for YLWHA currently available in schools, our study points to optimism and high potential given stakeholders’ identified avenues for improvement. We posit that promoting HIV/AIDS-care and support in schools is a gradual process requiring each school to develop a strong knowledge base about HIV/AIDS and support needs of YLWHA, develop a coherent and school-wide approach, and collaborate extensively with external stakeholders who are significant in supporting YLWHA.

**Supplementary Information:**

The online version contains supplementary material available at 10.1186/s12889-020-10143-3.

## Background

Schools ought to be inclusive spaces where all students can participate and belong irrespective of physical, cognitive, social, and emotional differences [1]. In order to attain inclusion for Youth Living with HIV/AIDS (YLWHA), a comprehensive education response involving the entire school structure (management, staff, students, and parents) is necessary [2]. The school staff also need to go beyond their academic role and take up pastoral and supportive care [2]. Such a response has been found to enhance resilience processes [3] and quality of life [4] in students living with HIV. While major challenges in terms of access to quality education for YLWHA and school staffs’ resources to address special needs are being observed in Sub-Saharan Africa (SSA) [5–7], studies position schools as effective support centres [8, 9]. Similarly, the Ugandan Ministry of Education and Sports’ national policy guidelines on HIV/AIDS promote inclusion of YLWHA in formal schooling and advocate for a multisectoral and multilevel approach to supporting the special needs of such students [10]. Supporting YLWHA in schools is particularly relevant as the prevalence of HIV continues to rise among youth in Uganda and other countries of sub-Saharan Africa [11] due to high infection rates during adolescence [12] and the scaling up of access to Antiretroviral Therapies (ART) [13]. Both perinatally and behaviorally infected young people are now growing into adulthood [14], necessitating a critical assessment of the HIV/AIDS response strategies of schools where this growing number of YLWHA is expected to spend most of the time [15].

Research on Ugandan secondary schools has shown that YLWHA are particularly vulnerable to HIV-related stigma, abuse, poor nutrition, mental and physical health difficulties, and poverty [6, 16–21]. Additionally, YLWHA often come from households that are economically challenged due to a high burden of healthcare-related costs and loss of income due to ill or deceased adult members [17]. As a consequence of the above, decline in school enrollment, attendance, and academic progress have been conspicuous effects of HIV among YLWHA, with significant consequences on other life domains too [17, 18, 22]. The contextual and multidimensional nature of the challenges outlined above requires a localized involvement of the key school stakeholders to spur support around such vulnerable students.

School-based HIV/AIDS interventions in countries of SSA have been systematically skewed towards primary prevention and are increasingly criticized for ignoring care and support (secondary and tertiary prevention) of the youth already infected [23]. This is similarly so with interventions by the Ministry of Education and Sports in Uganda, such as peer education clubs [24], drama and mass media campaigns [25], and the Presidential Initiative on AIDS Strategy for Communication to Youth programs [26]. Care and support initiatives for YLWHA have been mainly provided in environs external to schools, dominated by government, non-governmental organizations, and donor implementing partners, yet the bulk of the burden is borne by the immediate communities wherein YLWHA spend their daily lives [27]. The role of the schools in providing care and support to YLWHA as well as supporting externally driven interventions and policies has received little attention despite its potential for good outcomes [16].

In this study, employed and potential care and support strategies for YLWHA were explored with school stakeholders to identify advantageous avenues for promoting HIV/AIDS competent schools. Campbell and colleagues [27–29] define HIV/AIDS competent communities as social settings in which people are more likely to work collaboratively to prevent new HIV infections and to optimize care and support for those living with HIV/AIDS. Rooted in the socio-ecological perspective [30] and the concept of social capital [31] in health promotion, HIV/AIDS competence emphasizes interrelatedness and interdependence of individuals and their social environment for better health outcomes [32]. The HIV/AIDS competence framework is typified by six psycho-social resources whose presence or absence facilitates or hinders competence: knowledge and skills, safe social spaces, ownership and responsibility, confidence in local strength, solidarity, and bridging partnerships [28]. This framework provides for social settings to make right choices as intrinsic components of a durable and sustainable HIV/AIDS strategy [33]. While building HIV/AIDS competence is a long term process prone to hinderances such as HIV stigma [18], community tensions [34] as well as lack of knowledge, skills, and resources [35], evidence is available to suggest that HIV/AIDS competence yields positive results such as more care and support and improved quality of life of those afflicted [27]. We theorized that schools which deliberately develop care and support strategies for YLWHA have high potential of becoming HIV/AIDS-care and support competent schools .

Little is known about the current and probable future care and support strategies for YLWHA in Ugandan secondary schools. This study was motivated by the country’s aspirations of promoting inclusive education as a fulcrum for socio-economic development against the backdrop of schooling challenges for YLWHA expounded in the literature. We investigated what school stakeholders – i.e. key actors in the school supportive social structures- in secondary schools of western Uganda considered as advantageous strategies to care for and support YLWHA in their schools and how care and support could further be developed to realize inclusive education for YLWHA. The study was guided by the question ‘what do stakeholders (school staff, parents and students) consider as strategies to care for and support YLWHA in the schools?’

## Methods

### Study design, settings and participants

We conducted a qualitative exploratory inquiry using Focus Group Discussions (FGDs) and interviews, which were conducted in one district of western Uganda from May to October, 2019. FGDs were conducted in secondary schools and involved homogeneously composed school stakeholder groups (school staff, students and parents). We purposively selected three secondary schools that were accommodating both male and female students. We included one school from the urban, peri-urban, and rural settings respectively, with the anticipation that care and support strategies for YLWHA in these settings would differ due to differences in access to resources such as space, required number and categories of school staff, and finances. For each school, FGDs were conducted with representatives of each of the 3 stakeholder groups (student leaders, school staff with different leadership roles, and parents’ committee members). We purposively choose to engage these representatives because by virtue of their positions, they had a good view on the current and possible future strategies in their schools to care for and support YLWHA. Additionally, they are in a privileged position to rally and champion change within the schools. FGDs were also held with YLWHA aged 12–19 years selected from one ART clinic in a peri-urban setting. This group was selected from prior research contacts at this health facility and included members of a peer support group who were attending secondary school at that time though we were blinded to which school they were attending. After the FGDs with YLWHA, we sought the availability of their parents/caretakers to participate. Due to diversity in location of these parents/caretakers and lack of representation, we could not organize a focus group. We instead conducted individual interviews with these parents/caretakers. We reached these parents/caretakers by phone calls using contacts provided by YLWHA. One individual interview was conducted for each parent/caretaker at their homes or ART clinic as they preferred. We chose to engage YLWHA and their caretakers outside the school settings due to concerns of unintended HIV-status disclosure and stigma.

### Data collection

FGDs were conducted with 10 groups and involved a total of 83 participants with an average of 8 participants per group. At each school, we sought permission from the headteachers, who then selected a contact staff member to support the identification of potential participants. The staff member linked the research team with the heads of the different school stakeholder groups. After explaining the purpose and procedure of the study to these heads and obtaining their permission, they convened a group of their members with various leadership roles. All the 10 groups were involved in 2 consecutive sessions of FGDs with an interval of 1 or 2 days. In the first session, we explored the concern/challenges and resources/supports for YLWHA in school from the perspectives of these stakeholders. The stakeholders were also engaged in participatory ranking [36] to identify the hierarchy/priority of these challenges and supports. In the second session, participants were probed to reflect on what an ideal caring and supportive school environment would be for vulnerable students including those with HIV/AIDS and how their schools could be improved to accommodate the special needs of YLWHA. The identified strategies were also ranked and stakeholders were further supported to plan the future implementation of these strategies. Additional individual interviews reached data saturation with 5 parents/caretakers of school-going YLWHA. The interviews were based on the same topics as the FGDs.

Two researchers conducted the FGDs and interviews in quiet private places, using the same semi-structured guide, included as additional file 1. The researchers’ prior research experience in the schools and with YLWHA in these settings eased the process of reaching and engaging participants. No participant turned down the request to participate due to the trust that had been built. Each session of FGD lasted for averagely 110 min while interviews took about 75 min. FGDs and interviews were audio recorded with consent from participants. Note taking was also done to capture main ideas that arose as well as the non-verbal information. FGDs with students and school staff were conducted in English, an official language and medium of instruction in Ugandan schools. To avoid interrupting the learning and teaching, extracurricular time was utilized. FGDs with YLWHA were organized during school holiday and participants were encouraged to use a language of their choice. Interviews with parents and parents/caretakers of YLWHA were done on their ideal days and in the local language.

### Ethics

Ethical approval was obtained from Uganda National Council of Science and Technology (reference No. SS 4587) and the Institutional Review Boards of The AIDS Support Organization in Uganda (TASOREC/009/18-UG-REC-009) and Vrije Universiteit Brussel in Belgium (B.U.N 143,201,835,870). Informed written consent/assent was sought from YLWHA as well as the parents/caretakers of minors among YLWHA. All other participants provided verbal consent. Data were anonymized in the transcripts and reporting and were also confidentially kept. Parents/caretakers, school staff, and YLWHA were reimbursed for transport with the equivalent of 7 USD while all schools received games and sports materials as appreciation for participation of students.

### Data management and analysis

Data in audio files were transcribed verbatim by postgraduate students fluent in the languages used. FGDs and interviews conducted in local languages were translated to English and all transcripts were checked against the original audio recordings by the main researcher for accuracy. Thematic data analysis [37] was conducted involving inductive coding. The initial analysis involved a group of 5 authors: EK, SV, DR, AE and JD who independently read through 8 FGD and 2 interview transcripts to get familiar with the data and develop initial ideas to code. They then converged to discuss and harmonize these potential codes. The developed codebook was then used by EK to code all the transcripts in conformity with the research question, while remaining open to new emerging codes. Coding was done using the software NVivo version 10. Next, the codes together with all extracts of data that had been coded in respect to them, were collated into potential themes and overarching themes, most of which were deductively derived from the HIV/AIDS competence framework described in the introduction. EK and SV reviewed and refined the themes in an iterative way that led to collapsing, reordering, deletion and creation of themes. This ensured that data within themes was coherently meaningful, distinct from that in other themes and that the entire data breadth had been covered. Finally, we named the overarching themes, determined their essence, mapped their boundaries and established the intra-theme and inter-theme relationships to complete the analysis. Table 1 shows the overarching themes derived from the analysis.

**Table 1:** Themes derived from the analysis

Themes	Main themes
Peer counselling and guidance General counselling and guidance by teachers Professional counselling Spiritual counselling Sexuality and reproductive health counselling	Counselling and guidance
Peer friendships Clubs, games, and sports In-school mentors (‘guardian teachers’) Liaison with community resourceful people Liaison with nearby healthcare centres	Social support networks and linkages
Access to information Appropriate information about HIV/AIDS Sensitization of all school stakeholders Skills development Future prospects with schooling	Knowledge and skills
Non-discriminatory school policies Child protection policy Changing HIV/AIDS messages Attitude to normalize HIV/AIDS Rules and regulation against stigmatizing acts	Anti-stigma and anti-discrimination measures
Value of disclosure Partial disclosure Confidentiality and privacy Trust for disclosure Encouraging disclosure	Disclosure of HIV status
School nurses Essential medicines/first aid Sickbay Medication adherence support Referrals to healthcare centres	Treatment and management of HIV
Special meals for YLWHA Remedial classes Financial support	Affirmative action for YLWHA

### Findings

Care and support strategies for YLWHA in schools were located in 7 interrelated overarching themes that were derived through the analysis: counselling and guidance; social support networks and linkages; knowledge and skills; anti-stigma and anti-discrimination measures; disclosure of HIV status in school; treatment and management of HIV/AIDS; and affirmative actions for YLWHA. Since the current and proposed strategies were reported under the same themes, they are concurrently presented in the findings to minimize overlap and unnecessary repetitions.

### Counselling and guidance

In the Ugandan context, counselling and guidance is any form of advice given to a person with recognized or expected challenges to enable them surmount such challenges. A diagnosis of HIV creates a huge emotional load on YLWHA and as such, counselling and guidance is necessary.

Students reported providing peer counselling to those who disclosed to them on a friendship level. The school staff (teachers, school nurses, and matrons) provided general guidance and counselling to all students focusing on career growth, life skills, personal hygiene, general health, and Sexuality and Reproductive Health (SRH) noting that it was important to all students including YLWHA. They reported doing this periodically during school assemblies, in class, during extracurricular activities, and seldomly to support-seeking students. Only two school staff reported instances were YLWHA sought counselling from them.

In the urban school, teachers and students reported presence of a fulltime professional counsellor who was not part of the teaching staff and they stated that this allowed students with sensitive issues like HIV/AIDS to be counselled in privacy. In other schools, counselling was a duty of all school staff as quoted below;

*“Such students [YLWHA] get guidance and counseling from teachers. They learn how to live with HIV/AIDS, they also learn how to live with other people. We have many male and female teachers and they* [students] *are free to talk to any of their choice. So, you cannot say that you failed to get someone to talk to”* (female student, urban school).

Although this availed students opportunities to talk to a staff member of their choice, YLWHA cited non-confidentiality among teachers that often resulted into unintended disclosure. The school staff also noted that they had insufficient knowledge about HIV/AIDS and they lacked skills in HIV counselling as quoted below;

*“The school should have some counselors who can give the correct information to our children*

*whenever it is necessary. For us teaches we can provide some counseling but we may not*

*have the right messages to pass on to these youth [YLWHA]. It would also be necessary if health workers can visit teachers and train them on the right information for these youth [YLWHA] and how to give it. So, a supportive school environment should be one where every teacher is equipped to handle these unique cases that can occur in school” (Deputy headteacher, rural school).*

Students and school staff in all schools also deemed spiritual counselling supportive to YLWHA. Spiritual counselling emphasizes the role of God in providing divine strength and power for one to navigate challenges. It was reportedly provided by religious groups that occasionally visit the schools and also listen to concerns from individual students in a private arrangement.

One caretaker of a YLWHA reported providing SRH counselling to her daughter, stressing that such youth are highly vulnerable to sex abuse by men, which can lead to school dropout. All the other parents shied away from discussing SRH issues with their children, remarking that teachers were in the best position to do so.

In the rural school, the parents offered to support the school-based counselling programs by linking the school to their workmates and colleagues who are experts in HIV/AIDS counseling.

### Social support networks and linkages

Complex chronic health conditions like HIV/AIDS require support involving multiple individuals at different levels in society. Care and support strategies from social networks and linkages were prominently reported by participants.

All the stakeholders noted that their schools have diverse people from which YLWHA can make friends. Students and caretakers of YLWHA viewed friends of YLWHA as those who compassionately look out for them. Such friends were reported to provide material support, reminders to take medicines, and encouragement to YLWHA to stay in school. The two quotes below illustrate this support;

*“When I told my friends, they are the ones that now remind me to take my drugs. Sometimes they even bring drugs to me because they know where I keep it in my suitcase. They can even buy for me something to eat and say that ‘you need something to eat before taking your drugs’*

*They even make sure that I eat my food”* (18-year-old female YLWHA).

*“They [YLWHA] can make good friends at school. Some students are not rude, for example I know one of them who is very supportive to my daughter. She comes to visit her here and bring notes to her for the lessons she misses when sick. Also, the school nurse and matron have supported my daughter especially when she gets sick, they quickly tell me”* (female caretaker of a YLWHA).

As implied in the first quote, this support was realized when YLWHA disclosed their HIV status to the friends. As a way of promoting this support, most caretakers proposed to welcome friends of their HIV-positive youth in their homes and to treat them like their own children.

All stakeholder groups upheld the role of school clubs such as scripture union, peer education clubs, and interact clubs together with games and sports as avenues for YLWHA to make friends, interact, and share ideas. Additionally, clubs and games were reported to offer substantial distractions that prevent YLWHA from worrying about their status as quoted below;

*“Also, at school, they [YLWHA] get engaged in different activities like classwork, games, sports, clubs and they are always busy. Therefore, there is little time for them to keep worrying about their problems. They can even forget that they have that disease [HIV]”* (male student, urban school).

As a way of enhancing such distractive activities, students proposed formation of other supportive clubs for vulnerable students in which they would discuss their concerns. However, YLWHA in this study were against such clubs since they would evoke stigma but instead supported the idea of introducing various games, sports, and drama activities to encourage participation of all students. School staff and students proposed including indoor games like chess for students like YLWHA who they considered to be weak and unable to engage in field activities.

In the urban school, students alluded to presence of mentors. These were reportedly school staff designated to support students with unique challenges.

*“Okay, when you have a problem, at least everyone has a mentor. So, you can look for your mentor and talk to him or her. She can help you with your problem or connect you to the school administration in case where the administration has to come in”* (Female student, urban school).

However, YLWHA did not report benefiting from any mentorship program for their HIV/AIDS-related needs but for academic purposes. Students additionally suggested that introducing the ‘family’ concept at school would be beneficial to YLWHA. In such an arrangement, the school staff can take up the role of guardians to a group of students. In that ‘family’, challenges of individual members would be discussed and solutions sought. On the contrary, the other stakeholders were instead in support of promoting good linkages amongst themselves to create a network in which vital information can be shared to support YLWHA.

Beyond the immediate school stakeholders, other supportive linkages took the form of external counsellors that visited the schools as reported by YLWHA and caretakers of YLWHA. Parents also reported that the cooperation their schools had with community-based HIV/AIDS organizations was necessary for awareness talks and counselling in school. Although YLWHA preferred such external counsellors as already discussed, they were reviled for visiting schools in vehicles and attires that revealed them as people involved in HIV/AIDS care and support. YLWHA alluded to the involuntarily disclosure that this often caused. Students additionally proposed inviting community members who have lived with HIV/AIDS to share their experiences and encourage YLWHA.

In the peri-urban school, supportive linkages were created with the nearby healthcare centre. The quote below illustrates the kind of support this offers to YLWHA;

*“Another advantage we have is that we are near the health center IV and it is always open to us. We keep encouraging them* [students] *that if you have a health problem that you feel you do not want to disclose to us, then come and get permission and be escorted by the senior man or senior woman teacher to see a doctor at the healthcare centre. So, we take advantage of the health centre which is near the school”* (Male teacher, peri-urban school).

### Knowledge and skills

This theme presents participants’ views about their schools as sources of HIV/AIDS knowledge and a platform for socio-economic empowerment of YLWHA.

All students and school staff groups advocated for their schools as providing a good platform for YLWHA to learn more about their health condition. For instance, how to live longer, how to feed on a balanced diet and how to protect others. This is depicted in the quote below;

*“We also have a library which has a lot of materials. The school buys newspapers, we have*

*books on health which can be a source of information for these people* [YLWHA]*. We also have a television set in the school that they can watch. Sometimes there are talks on health on the TV” (*Female teacher, rural school).

YLWHA also emphasized that watching television at school availed them a lot of information about how to live with HIV through life stories.

All participants suggested that sensitization of all school stakeholders was necessary to raise knowledge about HIV/AIDS and for everyone to know their role in care and support for YLWHA. They further stated that through sensitization, appropriate information about HIV and those living with it can be provided, so that in-school support can be better tailored to the needs of YLWHA.

The students and school staff further reported that some messages in the school compound and those provided by teachers about HIV/AIDS were obsolete and needed to be changed. They gave examples of messages like, ‘HIV kills’, ‘avoid sex and avoid HIV’ which have been overtaken by current knowledge about HIV as quoted below;

*“The posters, okay that one is two-sided. I can say that they’re useful and sometimes they’re not. They’re useful to us who are not suffering from the disease* [*HIV/AIDS*]*. Of course, they’re trying to help you and to inform you that you should protect yourself so as not to get the disease* [*HIV]. And another thing, it’s bad to those who have the disease. Of course, they will be telling you that AIDS cannot be cured. Of course, you will neglect yourself, you’ll have that feeling that can even lead you to commit suicide”* (female student, urban school)*.*

### Anti-stigma and anti-discrimination measures

In this theme, we report participants’ current and suggested strategies to deal with HIV-related stigma and discrimination in their schools.

The school staff, students and parents stated that their schools do not discriminate those with HIV and hence all children are admitted and taught together despite their HIV status as illustrated in the quote below;

*“Teachers here, they always treat students equally. Because I have not yet seen any teacher*

*chasing out anyone suffering from HIV from class. Even on admission, they cannot say we are not going to admit you because you have HIV. So, they treat us equally”* (Female student, urban school)*.*

In the rural school, the school staff referred to their child protection policy that caters for the rights of all students including YLWHA.

As reported in preceding themes, regular counselling and activities for social interaction were also anti-stigma measure currently in schools that limit negative feelings in YLWHA.

Students and school staff in all schools suggested replacement of all stigmatizing messages in their school compound by more informative and empowering ones. Proposed alternatives messages were; “HIV is not the end of life”, “HIV should never determine one’s future”, “you can live a normal life with HIV”, and “HIV is a disease not a curse”, to tackle prevailing views of HIV/AIDS and the youth living with it. Together with the parents, students also suggested that stigmatizing language around HIV/AIDS should be avoided. They suggested that words like “HIV victims”, “people with the virus”, and “people on drugs” commonly used by fellow students and some school staff should be avoided. In the same vein, caretakers of YLWHA and YLWHA proposed that teachers should desist from using known and suspected YLWHA as examples in class while talking about HIV/AIDS.

All participants proposed instituting punitive measures for those who stigmatize and discriminate YLWHA. Rules and regulations against laughing at, abusing, teasing, mistreating, and unwarranted disclosing of YLWHA were proposed as illustrated bellow;

*“For me I think, there should be strict laws in school against abusing those who have HIV or*

*laughing at them in school and when others see that those who do it are punished, then that*

*habit can change. We can then motivate these students [with HIV] to work hard and be one of*

*the best in school. Therefore, others will look at them at the same level. Because if they are*

*performing very well then there will be no difference between them and other students”* (female student, rural school).

School staff additionally suggested that HIV stigma in the whole society should be addressed since what happens in schools is influenced by society in which school actors reside.

### Disclosure of HIV status in school

Revealing the status of people living with HIV voluntarily to others around them is encouraged by the HIV testing service policy for Uganda and it has been found to arouse support. In this theme, we report participants’ strategies to promote HIV-status disclosure and their views on benefits to YLWHA accruing from HIV-status disclosure.

Majority of the participants reported disclosure of YLWHA in school as necessary to provide adequate support as also reported in previous themes. Only two YLWHA in the study had disclosed their status and one caretaker of a YLWHA had disclosed the status of her child. The excerpt below illustrate the support that those who disclosed received;

*“The nurse keeps her medicine and reminds her every day to take it. You know my girl is very*

*careless. So, to ensure that her medicine is safe, she keeps it with the nurse. They [nurse and matron] also inform me when she is very sick and I have to pick her from school. Sometimes I just call them [nurse and matron] to ask how she is doing especially when she has been sick. They even look for her around school and I talk to her”* (female caretaker of a YLWHA).

The above quote further illustrates existing supportive social networks already discussed in the previous themes.

All participants proposed to promote partial disclosure but they differed regarding who YLWHA should disclose to. School staff and parents suggested disclosure to school administrators, perceiving them to be in a better supportive position as the quote below illustrates while students felt that it would be important for YLWHA to disclose to their peers.

*“We can encourage fellow parents with such students* [YLWHA] *in our parents meetings that if they have children who may need special attention then they should inform the school administration which can see how to support such children”* (Male parent, urban school).

YLWHA preferred to disclose to trustworthy people. One YLWHA even proposed that it would be better to disclose only to others like them [other YLWHA] since these have similar experiences and can provide the necessary support while maintaining secrecy.

To avoid involuntary disclosure which seemed detrimental to YLWHA, it was suggested that confidentiality and privacy should always be ensured. In the rural school, the school staff planned to develop a policy that would guide them on how to handle sensitive information about students in a confidential manner. Additionally, some YLWHA proposed that schools should provide a private room in which YLWHA can keep and take their medicine at school. The school staff and YLWHA suggested that teachers should check their suitcases and bags on reporting day at school in privacy instead of publicly as now often occurring, to prevent exposure of their ARV-drugs as typified in the quote below;

*“Also, during the checking of our school luggage when we go back to school, it should be done in privacy. I know they check everyone and we would not want to be checked in a separate place but everyone should be checked in privacy. When a teacher finds drugs in your case, he should not throw them around asking ‘what are these for?’ when everyone is there. Students should be checked one by one alone and not in a group. The teacher should not ask what are these? They should know how ARVs look like” (*16-year-old male YLWHA).

In the urban school, school staff suggested that teachers ought to create a conducive atmosphere that would encourage YLWHA to disclose by being more approachable, loving, caring and knowledgeable about HIV/AIDS.

### Treatment and management of HIV/AIDS

Living with HIV/AIDS requires lifelong treatment with ARVs in order to manage the HIV infection and prevent it from progressing to AIDS or to reverse AIDS. This however poses challenges for YLWHA within school. In this theme we explore strategies currently in schools and in future to support YLWHA to adhere to their treatment and rightly manage HIV/AIDS.

Some school staff, parents and caretakers of YLWHA stated that their schools had a designated staff such as a school nurse or a matron who was responsible for keeping and encouraging students to take their drugs as illustrated in the following quote;

*“Nurses have been able to seek HIV drugs for HIV victims [YLWHA]. Some students are shy to go there and get their drugs. So, nurses have been helping them to do that and we have a nurse who takes responsibility of those students in terms of getting drugs for them and encouraging them to take their drugs”* (school staff, rural school).

However, YLWHA noted that such individuals require training on the medical needs of YLWHA such as timely medication. One YLWHA stated that he always had to wait for the nurse at the time he was supposed to take his ARVs. Another, reported that the nurse was very rude and did not respect his privacy when giving him the drugs as this was often done in the presence of other students.

In other schools where nurses were not present, participants suggested that such essential staff should be included among school staff and they [nurses] should be adequately trained to handle unique medical conditions of students.

Some school staff, students and parents reported presence of essential drugs for common ailments such as cough, flue, headaches, fevers and stomach upsets in their schools. These were given as first aid to all students including YLWHA who they noted to be prone to such ailments. They suggested that this could be further improved if their schools put in place a well-equipped sickbay (a designated room for treatment of students) manned by a competent school nurse in which drugs can be kept and where the sick can take a rest while recovering. The sickbay was additionally foreseen to address the problem of privacy for students with HIV/AIDS to take drugs.

Parents and school staff in a rural school also suggested that more drugs such as ARVs could be added to the list of essential drugs that can be dispensed by nurses within the sickbay. The above concerns are illustrated in the quote below;

*“There should be a school nurse who is trained to cater for all students who may be HIV positive and others who may have other health conditions. Those who have problems should be able to access that nurse. In addition schools should have a well-equipped sick bay in which sick students can be taken care of by the nurse”* (school staff, rural school)*.*

In the urban school, the school staff and one caretaker of a YLWHA reported presence of a school van that transports sick students to the hospital for treatment. They noted that such an arrangement is available to YLWHA who often need to go for drug refills if they requested for it.

Treatment and management of HIV/AIDS was also reportedly supported by linkages and networks as already discussed. For instance, parents and teachers in the peri-urban school stated that their school refers students with health conditions they cannot manage to the nearby healthcare centre.

### Affirmative action for YLWHA

Given that HIV/AIDS imposes unique needs and challenges for those living with it and their families, in this theme we report strategies in schools that meet the unique academic and material needs of YLWHA.

In the peri-urban school, the school staff stated that they provide remedial classes for the students who miss class with genuine stated reasons as illustrated bellow;

*“We cater for individual differences. We cater for unique challenges that are communicated to us. For instance when a child stays away for a term because they are sick and the parent tells us, we allow that child back to school and we give them some remedial classes to catch up with the rest even when the parent has not told us what the child was suffering from”* (Female school staff, peri urban school).

The students supported this and further suggested that examinations and tests could also be given to known YLWHA if they miss them.

To address the financial hardships that YLWHA are prone to, school staff in the peri-urban school reported offering part-time jobs at school during holiday for those students who expressed their financial needs and were willing to work. However, all student groups proposed provision of bursaries and fee waivers for known students with HIV as presented in the quote below;

*“But bursaries are given to the best performing students who at time do not need any support*

*because their parents may be providing enough for them. Me I think that instead of giving*

*bursaries to the best performers, they should give them to such children* [YLWHA] so that they *can also be able to attain education*. *Because you may find that in their families, they are not able to cater for themselves”* (male student, peri-urban school).

Some school staff reported exempting known students with HIV/AIDS from strenuous activities such as cleaning and sports but YLWHA felt that this was discriminatory.

YLWHA proposed that schools should provide a balanced diet that includes vitamins and minerals necessary for YLWHA to build stronger defenses against the virus. Likewise, students suggested that school staff could link with parents of YLWHA to provide them with special meals at school but this was not supported by YLWHA due to the unintended disclosure it would cause.

## Discussion

We examined care and support strategies for YLWHA in Ugandan secondary schools from the perspectives of school stakeholders. We further sought their suggestions on how this care and support would further be developed in order to promote HIV/AIDS competent schools. Our findings are highly nuanced and defy simple summarization. They also show diversity in current and possible future strategies of various stakeholder groups and across settings. However, the findings portray limited current care and support specific for YLWHA but high potential in the different themes that we elaborated. Although different stakeholders reported and suggested several strategies, they often differed regarding the aptness, approach and leadership in such measures. We also found that some of the available and suggested strategies were counterproductive or unfeasible. From our findings, it appears that not only HIV-specific strategies provide care and support to YLWHA but also well executed general strategies that provide a safety net for all vulnerable students. Such strategies were reported across all the theme and include among others; first aid, school nurses and sickbays, fee waivers, nutritious meals, distractive activities, anti-discrimination measures, general counselling and friendships. Three features of HIV/AIDS competence [28] were most relevant to our findings: knowledge about HIV/AIDS and support needs of YLWHA; solidarity and common purpose in addressing needs of YLWHA; linkages with external supportive individuals and groupings. We examine each of these aspects in light of our findings and we provide recommendations.

Facilitating HIV/AIDS-care and support competence in schools requires a clear understanding of HIV/AIDS and the special needs of YLWHA. Severally, participants alluded to schools as the right settings in which such knowledge would be built. However, we found that school staff lacked adequate knowledge about HIV/AIDS care and support and as such felt incapable of delivering it to students. Quite often, the school staff blamed their laxity on non-disclosure, claiming that they cannot plan for a group whose existence is undercover. They instead located support for these students in settings external to school environs such as ART clinics and other community-based organizations. Considering the community readiness model [38], we hold that school communities by default need to be ready to receive and support YLWHA rather than instituting supportive measures following in-school disclosures. Since HIV is prevalent in this locality [6] and it is a highly stigmatized condition [18], schools should be at a high level of readiness regardless of any disclosures. Regrettably, our findings seem to place these schools at the level of vague awareness [39] in which they are concerned about the health issues – in this case care and support needs for YLWHA - but the motivation to address them is low. Most strategies that participants suggested would spur support and thus competence bordered on increasing knowledge about HIV/AIDS-care and support through training of teachers in HIV/AIDS counselling, reducing HIV stigma through regulations and changing language around HIV/AIDS to reflect current facts in HIV/AIDS-care and support. We view such strategies as significant in raising motivation and propelling the schools into next phases of action on the readiness continuum for building HIV/AIDS-care and support competence. As suggested by other scholars, schools should have factual knowledge of HIV/AIDS and sound awareness of all drivers of HIV-related stigma in their locality [40]. The knowledge would then enable them to gradually normalize and demystify HIV/AIDS [35]. Although we do not downplay the value of disclosure in determining the support provided or withheld from YLWHA, we hold a view that HIV/AIDS-care and support competent schools would discern such support needs a priori and create a welcoming environment wherein disclosure can easily occur. We thus recommend partial disclosure as YLWHA and their caretakers find it fit.

Due to their privileged social and technical position within schools that school staff (mainly teachers) possess [41], they were often placed at the frontline and expected to take a lead in caring for and supporting YLWHA. Although such leadership is necessary, highly competent communities bank on solidarity (mutual support) involving all stakeholders to build bonding social capital [42]. Our findings present individual labors of support by school staff, students and parents with less coordination and disharmony on what should be done and who should do it. As a result, some existing and suggested strategies seemed redundant, counterproductive or unattainable with regards to supporting YLWHA. For instance, students in one school proposed that YLWHA need to form a club in which issues affecting them can be discussed but YLWHA perceived this to elicit stigma. Also, school staff reported exempting known and suspected YLWHA from engaging in strenuous activities as a way of safeguarding their wellbeing but the beneficiaries viewed this as discriminatory. Building solidarity requires regular interface among the key players not only to discuss eminent and likely needs of YLWHA as they evolve but also how to jointly address them. It also involves apportioning responsibility as individual members are able to bear. For instance, teachers can develop local policies and guidelines against discrimination of YLWHA but student leaders can sensitize fellow students about discrimination and enforce the guidelines. In that case, ownership and responsibility, an essential aspect of HIV/AIDS competence is achieved.

The involvement of persons and agencies external to the school settings in support was extensively reported. Participants referred to visiting counsellors, visiting health workers and referrals to nearby health centers. Within the socioecological tenets [43], building HIV/AIDS-care and support competence entails strengthening such relationships with political, economic and technical power to facilitate effective school level response [29]. Such linking social capital [44] is foreseen to enhance bonding social capital described above through building knowledge, skills and confidence. For instance, farmers in the community can support the creation of vegetable gardens [45] to improve feeding in schools which several participants decried. The external counsellors that students and school staff clamored for can provide specialized training in HIV/AIDS counselling to teachers and address concerns of confidentiality and privacy so that students can receive adequate professional care as also proposed by Bhana and Morell [46]. Oftentimes, external agencies run out of resources since they rely on donor funds which are constrained by an array of societal needs and competing health priorities. In such instances, HIV/AIDS-care and support competent schools need to be effective support centres for YLWHA.

Lastly, since schools operate in a wider society within the policy and regulatory frameworks, the Ministry of Education should proactively support schools to build competence. As noted by Ainscow and colleagues [47], HIV/AIDS-specific supportive strategies require teachers to innovate ways of working. The remedial classes, private counselling sessions and sensitization of the wider community suggested by participants in this study are extra workloads requiring additional resources and new skills from teachers. If such strategies are not institutionalized or recognized, they remain to be implemented at the discretion of individual teachers and may not be sustained. While studying school response to child hardships in Kenya, Zimbabwe and Malawi, Kendall and O’Gara [48] found that teachers were overwhelmed and ill-equipped to deal with the emotional and psychosocial needs of children and thus recommended strategic investments by governments. We therefore propose an intersectoral approach in supporting the development of HIV/AIDS-care and support competent schools. Future research aimed at advancing HIV-care and support competent schools should also explore practicalities and potential challenges in the Ugandan context.

### Study strengths and limitations

Our findings are based on only responses from participants and therefore prone to selective reporting and recall biases. An ethnographic approach would have produced more credible data. However, we involved a wide range of participants that constitute school stakeholders together with schooling YLWHA and their parents. This enabled us to corroborate our finding and achieve reliability. We also did not involve policy makers such as local and national school governing authorities who are responsible for funding, supervising and appraising school performance. We believe that what occurs in schools is widely influenced by these bodies. We therefore recommend further research to explore those missed perspectives.

## Conclusions

Despite the limited care and support strategies specific for YLWHA currently available in schools, our study points to optimism and high potential. The findings challenge the assumption that schools are intrinsically well-placed to respond to extraordinary complex and multifaceted challenges in children. We argue that building HIV/AIDS-care and support competent schools is a gradual deliberate process requiring each school to develop a strong knowledge base about HIV/AIDS and support needs of YLWHA, rally solidarity within the school stakeholders to design a holistic approach since the strategies seem to be interdependent, and collaborate extensively with significant persons and organizations external to the school community. It also requires human and financial resources that are generated from within and without the settings.

## Supplementary Information

**Additional file 1.** Interview guide, used to conduct both focus group discussions and individual interviews.

## Data Availability

The dataset generated and analyzed during the current study is not publicly available because we did not seek consent from participants to share the data publicly. However, this dataset is available from the corresponding author on reasonable request.

## Abbreviations

AIDS: Acquired Immunodeficiency Syndrome
ART: Antiretroviral Therapy
FGD: Focus Group Discussion
HIV: Human Immunodeficiency Virus
SRH: Sexuality and Reproductive Health
USD: United States Dollar
YLWH: Youth Living With HIV/AIDS

## References

[1] Dixon S. Inclusion—Not segregation or integration is where a student with special needs belongs. The Journal of Educational Thought (JET)/Revue de La Pensée Éducative. 2005:33–53.

[2] UNESCO, I. (2008, November). Inclusive education: The way of the future. In Conclusions and recommendations of the 48th session of the International Conference on Education (ICE),(págs. 25–28). Geneva.

[3] Theron LC (2018). Teacher championship of resilience: Lessons from the pathways to resilience study, South Africa. Resilience in education.

[4] Wijngaarden, J., & Shaeffer, S. (2005). The Impact of HIV/AIDS on Children and Young People: Reviewing Research Conducted and Distilling Implications for the Education Sector in Asia. Discussion Paper No. I. UNESCO Bangkok. Asia and Pacific Regional Bureau for Education, PO Box 967, Prakhanong Post Office, Bangkok 10110, Thailand.

[5] Williams SE. Exploring the Viability of School-Based Support for Vulnerable Children: A Case Study of Two Township Schools in Johannesburg. CREATE Pathways to Access. Research Monograph No. 2010:46.

[6] Kimera, E., Vindevogel, S., De Maeyer, J., Reynaert, D., Engelen, A. M., Nuwaha, F., ... & Bilsen, J. (2019). Challenges and support for quality of life of youths living with HIV/AIDS in schools and larger community in East Africa: a systematic review. Systematic reviews, 8(1), 64. 10.1186/s13643-019-0980-1 PMC6390353 30808419

[7] Mitchell TD (2007). Critical service-learning as social justice education: A case study of the citizen scholars program. Equity & Excellence in Education.

[8] Ssewamala FM, Nabunya P, Mukasa NM, Ilic V, Nattabi J (2014). Integrating a mentorship component in programming for care and support of AIDS-orphaned and vulnerable children: lessons from the Suubi and Bridges Programs in Sub-Saharan Africa. Global Social Welfare.

[9] Shann MH, Bryant MH, Brooks MI, Bukuluki P, Muhangi D, Lugalla J, Kwesigabo G. The effectiveness of educational support to orphans and vulnerable children in Tanzania and Uganda. International Scholarly Research Notices. 2013;2013.

[10] Ministry of Education and Sports (2006). Education & sports sector national policy guidelines on HIV/AIDS. Accessed on 27th April 2020 via.

[11] Schuyler AC, Edelstein ZR, Mathur S, Sekasanvu J, Nalugoda F, Gray R (2017). & Santelli, J. S. Mobility among youth in Rakai, Uganda: Trends, characteristics, and associations with behavioural risk factors for HIV. Global public health.

[12] Schaefer, R., Gregson, S., Eaton, J. W., Mugurungi, O., Rhead, R., Takaruza, A., ... & Nyamukapa, C. (2017). Age-disparate relationships and HIV incidence in adolescent girls and young women: evidence from Zimbabwe. AIDS (London, England), 31(10), 1461. 10.1097/QAD.0000000000001506 PMC5457819 28426534

[13] Maartens G, Celum C, Lewin SR (2014). HIV infection: epidemiology, pathogenesis, treatment, and prevention. The Lancet.

[14] Mofenson LM, Cotton MF (2013). The challenges of success: adolescents with perinatal HIV infection. Journal of the International AIDS Society.

[15] Abubakar, A., Van de Vijver, F. J., Fischer, R., Hassan, A. S., Gona, J. K., Dzombo, J. T., ... & Newton, C. R. (2016). ‘Everyone has a secret they keep close to their hearts’: challenges faced by adolescents living with HIV infection at the Kenyan coast. BMC public health, 16(1), 197. 10.1186/s12889-016-2854-y PMC4772469 26927422

[16] Kimera E, Vindevogel S, Rubaihayo J, Reynaert D, De Maeyer J, Engelen AM, Bilsen J (2019). Youth living with HIV/AIDS in secondary schools: perspectives of peer educators and patron teachers in Western Uganda on stressors and supports. SAHARA-J: Journal of Social Aspects of HIV/AIDS.

[17] Kimera, E., Vindevogel, S., Kintu, M. J., Rubaihayo, J., De Maeyer, J., Reynaert, D., ... & Bilsen, J. (2020). Experiences and perceptions of youth living with HIV in Western Uganda on school attendance: barriers and facilitators. BMC public health, 20(1), 1–12. 10.1186/s12889-020-8198-7 PMC6969460 31952483

[18] Kimera, E., Vindevogel, S., Reynaert, D., Justice, K. M., Rubaihayo, J., De Maeyer, J., ... & Bilsen, J. (2020). Experiences and effects of HIV-related stigma among youth living with HIV/AIDS in Western Uganda: A photovoice study. *Plos one*, *15*(4), e0232359. 10.1371/journal.pone.0232359 PMC7182188 32330206

[19] Kembo J. Social and economic consequences of HIV and AIDS on children: case study of a high-density community in Harare, Zimbabwe. SAHARA-J: Journal of Social Aspects of HIV/AIDS. 2010;7(4). 10.1080/17290376.2010.9724976 21409310

[20] Mutumba M, Bauermeister JA, Musiime V, Byaruhanga J, Francis K, Snow RC, Tsai AC (2015). Psychosocial challenges and strategies for coping with HIV among adolescents in Uganda: a qualitative study. AIDS patient care and STDs.

[21] Birungi H, Obare F, Katahoire A, Kibenge D. HIV infection and schooling experiences of adolescents in Uganda. Social and Psychological Aspects of HIV/AIDS and their Ramifications. 2011:73–88.

[22] Kasirye I, Hisali E (2010). The socioeconomic impact of HIV/AIDS on education outcomes in Uganda: School enrolment and the schooling gap in 2002/2003. International Journal of Educational Development.

[23] Machawira P, Pillay V (2009). Writing in policy, writing out lives. Journal of Education Policy.

[24] Norton B, Mutonyi H (2007). ‘Talk what others think you can't talk’: HIV/AIDS clubs as peer education in Ugandan schools. Compare.

[25] Jacob WJ, Mosman SS, Hite SJ, Morisky DE, Nsubuga YK (2007). Evaluating HIV/AIDS education programmes in Ugandan secondary schools. Development in Practice.

[26] Stephen, N., & Costa, N. R. (2010). Educational Resources for HIV/AIDS Prevention in Uganda: The Role of PIASCY Program in Primary Schools. Journal of Educational Research (1027–9776), 13(1).

[27] Campbell, C., Scott, K., Nhamo, M., Nyamukapa, C., Madanhire, C., Skovdal, M., ... & Gregson, S. (2013). Social capital and HIV competent communities: the role of community groups in managing HIV/AIDS in rural Zimbabwe. AIDS care, 25(sup1), S114-S122. 10.1080/09540121.2012.748170 PMC3701935 23745625

[28] Campbell C, Nair Y, Maimane S (2007). Building contexts that support effective community responses to HIV/AIDS: a South African case study. American journal of community psychology.

[29] Campbell C, Foulis CA, Maimane S, Sibiya Z (2005). The impact of social environments on the effectiveness of youth HIV prevention: A South African case study. AIDS care.

[30] Golden SD, McLeroy KR, Green LW, Earp JAL (2015). & Lieberman.

[31] Hawe P, Shiell A (2000). Social capital and health promotion: a review. Social science & medicine.

[32] Latkin CA, Knowlton AR (2005). Micro-social structural approaches to HIV prevention: a social ecological perspective. Aids Care.

[33] Celletti, F., Wright, A., Palen, J., Frehywot, S., Markus, A., Greenberg, A., ... & Samb, B. (2010). Can the deployment of community health workers for the delivery of HIV services represent an effective and sustainable response to health workforce shortages? Results of a multicountry study. Aids, 24, S45-S57. 10.1097/01.aids.0000366082.68321.d6 20023439

[34] Gruber J, Caffrey M (2005). HIV/AIDS and community conflict in Nigeria: implications and challenges. Social Science & Medicine.

[35] Masquillier C, Wouters E, Mortelmans D, Van Wyk B (2015). On the road to HIV/AIDS competence in the household: building a health-enabling environment for people living with HIV/AIDS. International journal of environmental research and public health.

[36] Ager A, Stark L, Sparling T, Ager W (2011). Rapid appraisal in humanitarian emergencies using participatory ranking methodology (PRM). New York. NY.

[37] Braun V, Clarke V (2006). Using thematic analysis in psychology. Qualitative research in psychology.

[38] Plested BA, Edwards RW, Thurman PJ (2007). Disparities in community readiness for HIV/AIDS prevention. Substance use & misuse.

[39] Thurman PJ, Vernon IS, Plested B (2007). Advancing HIV/AIDS prevention among American Indians through capacity building and the community readiness model. Journal of Public Health Management and Practice.

[40] Skovdal M, Mwasiaji W, Webale A, Tomkins A (2011). Building orphan competent communities: experiences from a community-based capital cash transfer initiative in Kenya. Health policy and planning.

[41] Clarke DJ (2008). Heroes and villains: Teachers in the education response to HIV.

[42] Kawachi I, Berkman L. Social cohesion, social capital, and health. Social epidemiology. 2000;174(7).

[43] Bronfenbrenner U (1979). The ecology of human development. Harvard university press.

[44] Szreter S, Woolcock M (2004). Health by association? Social capital, social theory, and the political economy of public health. International journal of epidemiology.

[45] Ebersöhn L, Ferreira R (2011). Coping in an HIV/AIDS-dominated context: teachers promoting resilience in schools. Health education research.

[46] Bhana D, Morrell R (2006). The hidden work of caring: teachers and the maturing AIDS epidemic in diverse secondary schools in Durban. Journal of Education.

[47] Ainscow M, Dyson A, Weiner S (2013). From Exclusion to Inclusion: Ways of Responding in Schools to Students with Special Educational Needs. CfBT Education Trust. 60 Queens Road, Reading, RG1 4BS. England.

[48] Kendall N, O'Gara C (2007). Vulnerable children, communities and schools: lessons from three HIV / AIDS affected areas. Compare: A Journal of Comparative and International Education.

