# Supplementary material for: Care and support for youth living with HIV/AIDS in secondary schools: perspectives of school stakeholders in western Uganda
Source: BMC Public Health. 2021 Jan 6;21:63. doi: 10.1186/s12889-020-10143-3 (PMC7789575; doi:10.1186/s12889-020-10143-3)
Supplement: Supplementary file 1 — Additional file 1. Interview guide, used to conduct both focus group discussions and individual interviews. [file 12889_2020_10143_MOESM1_ESM.docx]

**Session 1 : Explorative study based on Focus Group and individual interview Method**

Present the research framework (objectives, rationale), situate the problem statement in relation to secondary schools referring to school drop-out or non-schooling of Youth Living With HIV/AIDS (YLWHA).

1. Explore major concerns/challenges for YLWHA in the school setting according to the stakeholders.
2. Explore good experiences and resources in place that support life quality of YLWHA at school.
3. Using participatory ranking, guide participants to rank the challenges and resources for YLWHA in their schools.

**Session 2 : Design study following a structured brainstorming method (e.g. GPS Brainstorm Kit)**

1. Out of the box thinking: what would a supportive school for all students (including those living with HIV/AIDS) ideally look like?
2. How can we improve this school setting so that it accommodates the special needs of students with HIV/AIDS?
3. On which of the barriers and supports can you intervene from your position? Which challenges are modifiable within the school context and what can you do about them to reduce them? Which supports are modifiable within the school context and what can you do to bolster/increase them?
4. What resources (time, finances, people,…) are needed to realize that? What are feasible strategies that you can start implementing from tomorrow onwards and that easily transferrable to other schools? (try to work from a master plan to a feasible strategy) Come up with an action plan and concrete strategies (to stipulate what will be done for whom, by whom with what resources in which time frame)

***Thank you very much for your time and response.***
